# Effect of Naturally Occurring Ozone Air Pollution Episodes on Pulmonary Oxidative Stress and Inflammation

**DOI:** 10.3390/ijerph120505061

**Published:** 2015-05-12

**Authors:** Cheryl Pirozzi, Anne Sturrock, Hsin-Yi Weng, Tom Greene, Mary Beth Scholand, Richard Kanner, Robert Paine

**Affiliations:** 1 Department of Internal Medicine, Division of Pulmonary and Critical Care Medicine, University of Utah, 26 North 1900 East, Salt Lake City, UT 84132, USA; E-Mails: Anne.sturrock@hsc.utah.edu (A.S.); Scholand@genetics.utah.edu (M.B.S.); Richard.kanner@hsc.utah.edu (R.K.); Robert.Paine@hsc.utah.edu (R.P.III); 2 Department of Pediatrics, University of Utah, Salt Lake City, UT 84132, USA; E-Mail: cindy.weng@hsc.utah.edu; 3 Department of Internal Medicine, Division of Epidemiology, University of Utah, Salt Lake City, UT 84132, USA; E-Mail: Tom.Greene@hsc.utah.edu

**Keywords:** air pollution, ozone, chronic obstructive pulmonary disease, exhaled breath condensate, oxidative stress, airway inflammation

## Abstract

This study aimed to determine if naturally occurring episodes of ozone air pollution in the Salt Lake Valley in Utah, USA, during the summer are associated with increased pulmonary inflammation and oxidative stress, increased respiratory symptoms, and decreased lung function in individuals with chronic obstructive pulmonary disease (COPD) compared to controls. We measured biomarkers (nitrite/nitrate (NO_x_), 8-isoprostane) in exhaled breath condensate (EBC), spirometry, and respiratory symptoms in 11 former smokers with moderate-to-severe COPD and nine former smokers without airflow obstruction during periods of low and high ozone air pollution. High ozone levels were associated with increased NO_x_ in EBC in both COPD (8.7 (±8.5) *vs.* 28.6 (±17.6) μmol/L on clean air *vs.* pollution days, respectively, *p* < 0.01) and control participants (7.6 (±16.5) *vs.* 28.5 (±15.6) μmol/L on clean air *vs.* pollution days, respectively, *p* = 0.02). There was no difference in pollution effect between COPD and control groups, and no difference in EBC 8-isoprostane, pulmonary function, or respiratory symptoms between clean air and pollution days in either group. Former smokers both with and without airflow obstruction developed airway oxidative stress and inflammation in association with ozone air pollution episodes.

## 1. Introduction

Ground level ozone air pollution is associated with adverse health effects and may have important health consequences for individuals with chronic obstructive pulmonary disease (COPD). Long-term exposure to ozone has been linked with increased respiratory mortality [1] and increased mortality among persons with COPD [2]. Short-term exposure to ambient ozone pollution is associated with decreased lung function [3] and increased hospitalizations for COPD [4,5,6,7]. Thus, episodes of ozone air pollution exposure likely represent significant stress episodes for individuals with COPD. However, it is unclear to what extent those individuals respond differently to ozone pollution compared to controls.

The pathophysiologic mechanisms resulting in the clinical response to air pollution are poorly defined, but are likely similar to the underlying pathophysiology of COPD itself, and involve oxidative stress and local inflammation in the lung. Multiple components of outdoor air pollution are sources of oxidative stress, which may induce lung damage and contribute to disease progression in COPD [8,9]. These mechanisms may result in a fundamentally different response to outdoor air pollution episodes in individuals with COPD compared to those without lung disease.

Exhaled breath condensate (EBC) is a noninvasive method of sampling the airway lining fluid to analyze changes in the local pulmonary environment. This approach has shown promise for identifying biomarkers indicating pulmonary inflammation and oxidative stress. Multiple markers of oxidative stress and inflammation are increased in EBC of COPD patients, including nitrite + nitrate (NO_x_) [10,11] and 8-isoprostane [12,13]. Exposure to high levels of ambient particulate air pollution has been associated with increased markers of pulmonary inflammation and oxidative stress [14,15]. It has not yet been determined if increased levels of ozone air pollution are associated with increased EBC biomarkers of inflammation and oxidative stress in patients with COPD or if the response to air pollution episodes differs between individuals with COPD and controls without COPD.

The Salt Lake Valley in Utah, USA, experiences elevated levels of ozone air pollution during the summer with levels exceeding the Environmental Protection Agency (EPA) National Ambient Air Quality Standards (NAAQS). These relatively predictable air pollution episodes provide an opportunity to investigate the impact of naturally occurring elevated ambient ozone on individuals with COPD compared to appropriate controls.

We hypothesized that increased levels of ozone air pollution would be associated with increased pulmonary inflammation and oxidative stress, indicated by increased EBC biomarkers, as well as increased respiratory symptoms and decreased lung function. Furthermore, we hypothesized that this response would be exaggerated in individuals with COPD compared to former smokers without COPD.

## 2. Methods

We conducted a prospective observational study comparing characteristics of EBC biomarkers, spirometry, and respiratory symptoms in COPD and control subjects under naturally occurring conditions of good and poor air quality. All participants were adults aged 40–85 living in the Salt Lake Valley in Utah. The COPD group consisted of former smokers with moderate or severe airflow obstruction [16]. The control group consisted of former smokers without overt chronic lung disease, airflow obstruction, or emphysema on CT imaging. Non-smoking status was by identified by self-report and verified by exhaled carbon monoxide levels in 19 of 20 participants. Inclusion and exclusion criteria are summarized in Table 1. Subjects were recruited from the Pulmonary Clinic and Pulmonary Function Test Lab of the University of Utah, and the Lung Health Research Center at the University of Utah. As this was a pilot study, the sample size was based on the number of individuals identified who met entry criteria between January and June 2012 and who agreed to participate. Approval was obtained from the University of Utah Institutional Review Board.

**Table 1:** Inclusion and exclusion criteria.

Inclusion Criteria		Exclusion Criteria
COPD Group	Control Group	All Groups
Former smoker	Former smoker	Active smoking
≥10 pack year smoking history, quit at least 3 months prior to enrollment	≥10 pack year smoking history, quit at least 3 months prior to enrollment	Any significant pulmonary disease other than COPD which would limit the interpretability of the pulmonary function measures
Age 40–85	Age 40–85	COPD exacerbation * in the prior six weeks
Moderate or severe COPD (FEV1/FVC below the lower limit of normal and FEV1 <70% predicted for age and height)	Spirometry without evidence of airflow obstruction (FEV1/FVC greater than the lower limit of normal)	Currently taking ≥10 mg a day of prednisone or equivalent systemic corticosteroid
	No evidence of emphysema on CT imaging, if previously obtained	Inability to perform exhaled breath condensate, spirometry, or complete respiratory symptom questionnaire
		Pregnant or intending to become pregnant

* Note: An acute exacerbation of COPD was defined as a sustained worsening of the patient’s condition, from the stable state and beyond normal day-to-day variations, that is acute in onset and necessitates a change in regular medication in a patient with underlying COPD [17].

The study took place during the summer, June–September 2012*.* Participants completed a baseline questionnaire regarding residential history, exposure to smoke, pollution, or occupational exposures, and disease history.

Participants were evaluated both during periods of good air quality and in “triggered visits” initiated during periods of poor air quality based on measurements of 8 h ozone updated hourly from the Salt Lake City Hawthorne Station, which is the controlling monitor for the Salt Lake Valley. Good air quality periods were defined by an 8 h ozone level ≤0.059 ppm for ≥4 consecutive preceding days. The Utah Department of Environmental Quality Division of Air Quality defines “Red Alert” days by an 8 h ozone level >0.075 ppm, which is the threshold value which exceeds the National Ambient Air Quality Standards (NAAQS). “Yellow Action” days are defined by an 8 h ozone level 0.068 ppm–0.075 ppm [18]. Poor air quality testing days included those with 8 h ozone level >0.075 ppm, or 2 consecutive or 3 out of 4 days with 8 h ozone ≥0.068 ppm. We incorporated a 0–1 day lag for testing on poor air quality days based on the 0–1 day lag-effect on symptoms, mortality, and lung function seen in prior studies with ozone pollution [3,19,20,21,22]. Based on the air quality forecast from the Utah Division of Air Quality, participants were contacted and asked to come to the study center during periods of poor air quality, and testing was carried out if ozone levels indeed met the predetermined levels for poor air quality. Testing on good air quality days occurred after there had been a minimum of 4 days of consecutive good air quality after a period of poor air quality.

Testing at each visit included EBC collection for biomarker analysis, spirometry, and completion of a respiratory symptom questionnaire. EBC was used to measure NO_x_ and 8-isoprostane as biomarkers of oxidative stress and inflammation. EBC was collected at each visit using the R-tube system [23] according to standard protocol with tidal breathing for 10 min. Approximately 1–2 mL condensate fluid was collected from each participant. Samples were divided into 200 μL aliquots and frozen at −80 degrees F. EBC NO_x_ was measured using the colorimetric Griess enzymatic reaction with Total Nitric Oxide and Nitrate/Nitrite Parameter Assay Kit (R&D Systems). 8-isoprostane was measured by ELISA using Cell Biolab OxiSelect™ 8-iso-Prostaglandin F2a ELISA Kit. Spirometry without bronchodilator was conducted at each study visit according to ATS criteria. Subjects continued all home medications prior to testing except they were asked to hold short-acting beta agonists for 4 h prior to spirometry if able.

Respiratory symptoms were assessed using a questionnaire that assessed change from baseline in eight symptoms over the preceding few days: shortness of breath, sputum thickness or color, amount of sputum, cough, wheeze, chest tightness, nasal congestion or discharge, and feeling of activity limitation due to lung condition (Table 2). Symptoms were analyzed as the total aggregate score obtained by adding the individual symptom scores.

**Table 2:** Respiratory symptom questionnaire.

Symptoms assessed	Shortness of breath	
	Sputum thickness or color	
	Amount of sputum	
	Cough	
	Wheeze	
	Chest tightness	
	Nasal congestion or discharge	
	Feeling of activity limitation due to lung condition	
Response choices for each symptom	Change from baseline	Score
	Symptoms have decreased	0
	Symptoms are the same	1
	Symptoms have increased a little	2
	Symptoms have increased a lot	3
	I don’t know or I don’t experience this symptom	-

### Statistical Methods, Data Analysis and Interpretation

The primary outcomes were 8-isoprostane and NO_x_ in exhaled breath condensate. Secondary outcomes included FEV1, FVC, and respiratory symptoms.

Baseline characteristics were summarized for the COPD and control cohorts. Baseline characteristics were compared between the COPD and former-smoker control groups using 2-sample *t*-tests for quantitative variables.

The lung function, inflammatory, and oxidative stress outcomes were analyzed using separate linear mixed effect models with random effects for each patient and a fixed effect to distinguish between pollution and clean air days to estimate the mean differences between pollution and clean air days within the COPD and former smoker groups, respectively. We then applied a mixed model including both the COPD and control groups with random effects for each individual and fixed effects to designate the COPD and control groups, the pollution *vs.* clean air assessments to estimate the difference in estimated pollution effect (compared with clean air days) between the COPD and control groups. We applied mixed effects models in order to incorporate all available lung function, inflammatory, and oxidative stress measurements in a statistically efficient manner even when the numbers of visits differed between the pollution and clean air days [24]. The model for the FVC incorporated different residual variances for the COPD and control groups as a likelihood ratio test indicated a significantly higher level of variability for the COPD group. The aggregate symptom score was analyzed using the same mixed effects models used for the quantitative outcomes. Frequencies and proportions of patients experiencing a worsening of symptoms on at least one pollution day and on at least one clean air day were also summarized.

The analyses of this observational study were interpreted as exploratory, and results were regarded as statistically significant using a 2-sided significance level of 0.05, without adjustment for multiple comparisons. All analyses were performed used SAS 9.4 (SAS 9.4, SAS Inc., Cary, NC, USA).

## 3. Results

### 3.1. Study Subjects

We enrolled 11 former smokers with moderate to severe COPD (four with moderate and seven with severe to very severe airflow obstruction) and nine former smoker controls without airflow obstruction. Baseline characteristics are shown in Table 3. The COPD group had greater mean pack-years smoking history, and fewer years since quitting smoking. As expected, the COPD group had airflow obstruction on spirometry and demonstrated lower mean FEV1, FVC, and FEV1/FVC.

**Table 3:** Baseline characteristics.

	COPD [55% (*n* = 11)]	Control [45% (*n* = 9)]	*p*-Value
Male [% (*n*)]	72.7% (8)	55.6% (5)	0.64
Age [mean (±SD)]	70.7 (±4.7)	66.8 (±5.6)	0.11
Smoking history [mean (±SD)]			
Smoking (pack-years)	77.7 (±27.7)	41.3 (±18)	<0.01
Years since quitting smoking	6.6 ( ±6.4)	15.4 (±6.5)	<0.01
Spirometry [mean (±SD)]			
FEV1 (L)	1.2 (±0.6)	2.5 (±0.4)	<0.01
FEV1 % predicted	41.3 (±17.4)	89 (±11)	<0.01
FVC (L)	2.9 (±0.9)	3.3 (±0.7)	0.21
FVC % predicted	73.2 (±13.9)	88.5 (±10.9)	0.01
FEV1/FVC %	41.1 (±14.3)	75.6 (±5.1)	<0.01
Other baseline characteristics [% (*n*)]			
Second hand smoke exposure	100% (11)	88.9% (8)	0.45
History of asthma	18.2% (2)	11.1% (1)	1.00
History of VGDF exposure	9.1% (1)	11.1% (1)	1.00
Taking inhaled long acting anticholinergic	72.7% (8)	0	<0.01
Taking inhaled short acting beta agonist	72.7% (8)	11.1% (1)	0.01
Taking inhaled corticosteroid	72.7% (8)	11.1% (1)	0.01
Taking inhaled long acting beta agonist	72.7% (8)	11.1% (1)	0.01

Notes: *p*-Values for comparisons between the COPD and Controls were computed using t-tests for continuous variables and Fisher exact tests for categorical variables (gender, history of antibiotics, and use of inhaled medications. VGDF = vapors, gases, dusts, or fumes.

### 3.2. Air Quality

**Figure 1:**
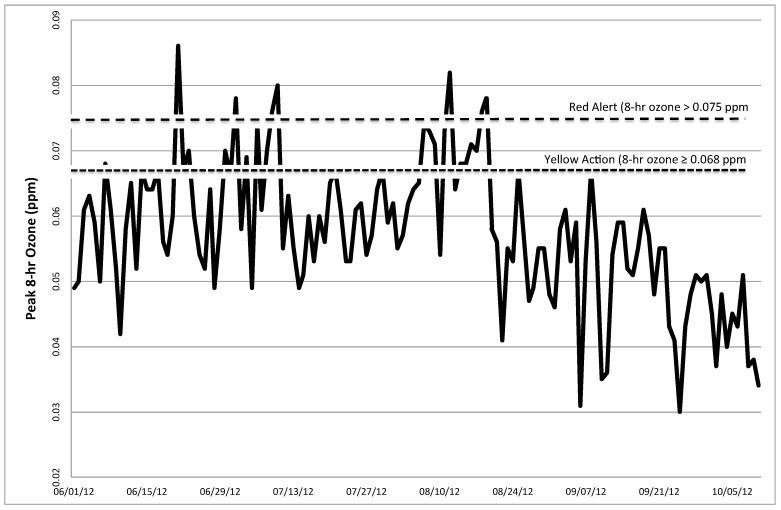
Daily peak 8-h ozone in Salt Lake Valley, UT during Summer 2012. Dashed lines indicate the level of 8-h ozone designated as “Red Alert” and “Yellow Action” days by the Utah Division of Air Quality. “Red Alert” days are defined by an 8 h ozone level >0.075 ppm, which is the value which exceeds the National Ambient Air Quality Standards (NAAQS). “Yellow Action” days are defined by an 8 h ozone level ≥0.068 ppm. Poor air quality testing days included those with 8 h ozone level >0.075 ppm, or 2 consecutive or 3 out of 4 days of 8 h ozone ≥0.068 ppm.

Mean maximum pollutant levels were calculated by averaging the day of testing and one day prior. Mean (±SD) peak 8 h ozone was 0.046 (±0.01) ppm on clean air testing days, and 0.067 (±0.01) ppm during pollution testing days. Figure 1 shows the trends of daily maximum 8-h ozone in Salt Lake County during the summer and early fall of 2012. Peak levels of other criteria pollutants remained well below the EPA NAAQS during the period of our study. In particular, mean (±SD) peak levels of 24-h PM_2.5_, 1-h nitrogen dioxide (NO_2_), 1-h sulfur dioxide (SO_2_), and 1-h carbon monoxide (CO) were 9.3 (±4.9) μg/m^3^, 0.034 (±0.004) ppm, 2.73 (±1.71) ppb, and 0.88 (±0.26) ppm respectively, on clean air testing days and 9.2 (±4.6) μg/m^3^, 0.028 (±0.010) ppm, 1.47 (±1.00) ppb, and 0.53 (±0.22) ppm, respectively, on pollution testing days (Table 4). Of the COPD participants, seven had one visit and four had two visits on poor air quality days, and nine had one visit on good air quality days. Of the former smoker controls, six had one visit and two had two visits on poor air quality days, and eight had one visit on good air quality days.

**Table 4:** Pollutant levels on clean air and pollution testing days.

Pollutant	Clean Air Testing Days	Pollution Testing Days
Ozone (8-h)	0.046 (±0.01) ppm	0.067 (±0.01) ppm
PM_2.5_ (24-h)	9.3 (±4.9) μg/m^3^	9.2 (±4.6) μg/m^3^
NO_2_ (1-h)	0.034 (±0.004) ppm	0.028 (±0.010) ppm
SO_2_ (1-h)	2.73 (±1.71) ppb	1.47 (±1.00) ppb
CO (1-h)	0.88 (±0.26) ppm	0.53 (±0.22) ppm

Notes: Displayed are mean (±SD) peak levels of measured pollutants on clean air and pollution testing days. PM_2.5_ = particulate matter less than 2.5 microns in diameter; NO_2_ = nitrogen dioxide; SO_2_ = sulfur dioxide; CO = carbon monoxide; SD = standard deviation.

### 3.3. Exhaled Breath Condensate Parameters

**Figure 2:**
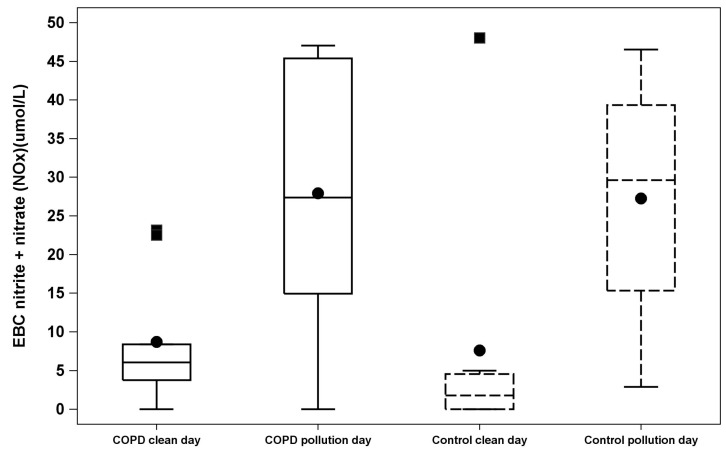
Exhaled breath condensate (EBC) nitrite + nitrate (NO_x_) between pollution and clean air testing days. Box-and-Whisker plots shows the distribution of exhaled breath condensate (EBC) nitrite + nitrate (NO_x_) between pollution and clean air testing days classified by COPD *versus* Control groups. Black filled dots represent mean of EBC nitrite + nitrate (NO_x_). Black filled squares represent the outliers (above maximum value). The “whisker” represents minimum and maximum values. The box plot goes from upper (75) percentile, median and lower (25) percentile of the values.

We found evidence of pollution-related increases in airway inflammation and oxidative stress in EBC from subjects with COPD and controls, without significant difference between the two groups in their response to pollution. Elevated ozone air pollution was associated with increased EBC NO_x_ both in participants with COPD and in control participants (COPD mean level 8.7 (±8.5) *vs.* 28.6 (±17.6) μmol/L on clean air *vs.* pollution days, respectively, estimate of difference for clean air *vs.* pollution days 20.7, 95% CI 7.93 to 33.47, *p* = 0.004, and control subject mean level 7.6 (±16.5) *vs.* 28.5 (±15.6) μmol/L on clean air *vs.* pollution days, respectively, estimate of difference 20.1, 95% CI 3.88 to 36.35, *p* = 0.02) (Figure 2, Table 5). The pollution effect on EBC NO_x_ did not differ significantly between the COPD and control groups (*p* = 0.94). Conversely, ozone pollution days were not significantly associated with changes in EBC 8-isoprostane in either group.

**Table 5:** EBC biomarker results on clean air quality *vs.* pollution days.

EBC Biomarker	Comparisons	Clean Air Day Mean (±SD)	Pollution Day Mean (±SD)	Estimate of Difference	95% CI	*p*-Value
Nitrite + nitrate (NO_x_) (umol/L)	COPD Clean *vs.* Pollution Day	8.7 (±8.5)	28.6 (±17.6)	20.7	7.9 to 33.5	<0.01
	Control Clean *vs.* Pollution Day	7.6 (±16.5)	28.5 (±15.6)	20.1	11.2 to 29.9	0.02
	Pollution effect COPD *vs.* Control			0.63	−18.1 to 19.4	0.94
EBC 8-isoprostane (pg/mL)	COPD Clean *vs.* Pollution Day	5 (±5.6)	6.9 (±8.8)	1.99	−4.7 to 8.7	0.53
	Control Clean *vs.* Pollution Day	12.3 (±9.7)	12.8 (±8.9)	2.87	−1.2 to 7.0	0.14
	Pollution effect COPD *vs.* Control			0.62	−8.3 to 9.5	0.88

Notes: Displayed is estimated difference from mixed effect model (with 95% CIs and *p*-values) of 8-isoprostane and NO_x_ between pollution and clean air days in (a) COPD patients and (b) Control group patients, as well as (c) the estimate of difference which compares the estimated pollution effects from (a) and (b) between the COPD patients and Controls.

### 3.4. Clinical Parameters

We did not detect a significant difference in pulmonary function between pollution and clean air days in either group (Table 6). Similarly, respiratory symptoms were not increased on ozone pollution days compared with clean air days in COPD patients (difference in aggregate symptom score −0.85, CI −3.54 to 1.83, *p* = 0.50) or control subjects (difference in aggregate symptom score −1.09, CI −4.34 to 2.16, *p* = 0.44) (Table 6, Table 7). The pollution effects did not differ between the two groups (*p* = 0.88).

**Table 6:** Spirometry and aggregate symptom score on pollution *vs.* clean air quality days.

Variables	Comparisons	Difference	95% CI	*p*-Value
FEV1 (L)	COPD pollution *vs.* clean days	0.01	−0.11 to 0.13	0.86
	Control pollution *vs.* clean days	−0.07	−0.17 to 0.03	0.15
	Pollution effect COPD *vs.* Control	0.08	−0.076 to 0.24	0.29
FVC (L)	COPD pollution *vs.* clean days	0.04	−0.18 to 0.26	0.69
	Control pollution *vs.* clean days	−0.11	−0.23 to 0.01	0.08
	Pollution effect COPD *vs.* Control	0.15	−0.12 to 0.42	0.26
Aggregate symptom score	COPD pollution *vs.* clean days	−0.85	−3.54 to 1.83	0.50
	Control pollution *vs.* clean days	−1.09	−4.34 to 2.16	0.44
	Pollution effect COPD *vs.* Control	0.29	−3.66 to 4.24	0.88

Notes: Displayed are estimated mean differences (with 95% CIs and *p*-values) in FEV1, FVC, and aggregate symptom score between pollution and clean air days in (a) COPD patients and (b) Control group patients, as well as (c) the difference in the estimated pollution effects from (a) and (b) between the COPD patients and Controls.

**Table 7:** Percent of patients reporting worsening symptoms on pollution or clean air quality visits.

Symptoms	COPD		Control	
	Clean Air Days	Pollution Days	Clean Air Days	Pollution Days
	% (*n*)	% (*n*)	% (*n*)	% (*n*)
Nasal congestion discharge	33.33 (3)	36.36 (4)	37.5 (3)	12.5 (1)
Activity limitation	33.33 (3)	54.55 (6)	12.5 (1)	0 (0)
Chest tightness	11.11 (1)	27.27 (3)	0 (0)	25 (2)
Cough	11.11 (1)	27.27 (3)	37.5 (3)	37.5 (3)
Shortness of breath	22.22 (2)	36.36 (4)	0 (0)	0 (0)
Sputum thickness	22.22 (2)	18.18 (2)	25 (2)	37.5 (3)
Sputum amount	33.33 (3)	27.27 (3)	25 (2)	37.5 (3)
Wheeze	11.11 (1)	18.18 (2)	12.5 (1)	12.5 (1)

Notes: Displayed are the number and percent of patients reporting worsening symptoms on at least one polluted air day and on at least one clean air day for the Control and COPD groups.

## 4. Discussion

We found that in former smokers, exposure to episodes of ozone pollution was associated with an increase in NO_x_ in exhaled breath condensate. There was no difference in effect between those with and without airway obstruction. Despite the change in EBC biomarkers, we did not detect a change in pulmonary function or respiratory symptoms in response to these pollution events. These findings support the hypothesis that environmental ozone air pollution exposure results in increases in airway inflammation and oxidative stress in individuals who are former smokers; however we did not detect an exaggerated response in individuals with COPD compared to former smokers without COPD.

To our knowledge, this study is the first to demonstrate an association of naturally occurring short-term ozone air pollution exposure with increased EBC NO_x_ in former smokers with and without COPD. Experimental controlled exposure of healthy volunteers to high levels of ozone was associated with increased EBC NO_x_ in one study [25] but not in others [26,27]. In other studies, short-term exposure to other naturally occurring particulate and gaseous pollutants has been associated with increased EBC markers of inflammation and oxidative stress in adults with chronic respiratory disease including COPD [15], healthy adults [14], and children with asthma [28,29]. EBC is attractive as a window into the local environment in the peripheral lung because it is noninvasive and easily repeated. A number of different biomarkers have been described in EBC. We focused on total EBC concentrations of NO_x_ and 8-isoprostane as validated measures of inflammation and local oxidative/nitrosative stress in the airways in COPD patients [14,15]. Manney *et al.* (2012) found increases in EBC NO_x_ associated with exposure to coarse particles, and Huang *et al.* (2012) found increases in EBC NO_x_ associated with exposure to particulate pollution, elemental carbon, SO_2_, CO, and NO_2_, but not with exposure to ozone air pollution. A few prior studies have examined effects of ozone air pollution on exhaled breath condensate parameters. Both Liu *et al.* (2009) and Barraza-Villareal *et al.* (2008) examined effects of ozone exposure on asthmatic patients; Liu *et al.* did not find an association of ozone exposure with EBC biomarkers and Barraza-Villarreal *et al.* found an association of ozone with EBC IL-8. Neither measured EBC NO_x_.

There are several important features of this pilot study. The recurrent wide swings in ambient ozone levels to well above the EPA National Ambient Air Quality Standards that occur in the Salt Lake Valley during the summer offer a unique natural laboratory to study the effects of short-term air pollution exposure. Our measurement of biomarkers in exhaled breath condensate, in conjunction with respiratory symptoms and lung function, offers insight into the pathophysiology of observed clinical associations. Increased NO_x_ in exhaled breath condensate suggests that exposure to ozone air pollution activates inflammatory and oxidative/nitrosative stress pathways in the airways, and that this may be the mechanism for changes in respiratory health, including hospitalizations for COPD, observed in numerous prior studies.

A key aspect of this study was the selection of former smokers for both the COPD and control groups. This choice avoided confounding effects of recent cigarette use on parameters in EBC. It also offered an opportunity to compare responses to air pollution between smokers who had developed COPD and those who had not. It remains unclear why only a minority of smokers develop clinically significant airflow obstruction and host characteristics could affect susceptibility to developing COPD as well as response to environmental exposures such as air pollution. However, we did not find significant differences in airway inflammation induced by ozone air pollution episodes between former smokers who had developed COPD and former smokers without airflow obstruction. This suggests that exposure to ozone air pollution triggers airway inflammation in former smokers regardless of certain host characteristics such as airway obstruction. Other studies have found association of ozone pollution and health effects in normal subjects without a greater effect in participants with COPD [3,19]. Several studies have demonstrated an association of short-term ozone exposure with increased daily mortality in large populations [20,30,31,32,33] and without increased mortality risk in individuals with COPD [19,34,35]. It is possible that our findings reflect increased susceptibility to inflammation in former smokers and a comparison with never smokers might have yielded different results, however studies have shown baseline EBC NO_x_ in stable COPD to be comparable to levels in healthy never smokers as well as similar levels between healthy smokers and nonsmokers [10,36].

Unlike prior studies, we did not find a significant association of short-term ozone air pollution exposure with increased respiratory symptoms [37] or decreased lung function [3]. Our study may have been too small to detect symptom or lung function changes. EBC NO_x_ may be a more sensitive indicator of physiologic stress than clinical change. It is also possible that clinical change occurs after a lag time that was outside our testing window or that the actual duration of outdoor activity for the subjects in this study was too brief to induce changes in respiratory symptoms. In addition, daily spirometry might detect more subtle changes and elucidate timing of changes in respiratory function after exposure to ozone.

There are several limitations to this study. We studied a small sample size of Caucasian, middle and older aged adults in a single small geographic area. The control and COPD groups differed in extent of smoking history. Due to the small sample size, confidence limits for comparisons of comparisons of pollution *vs.* clean air days were wide, and we are not able to rule out undetected effects in cases where comparisons were not statistically significant. Individual pollution exposure was estimated based on average ozone level from a central measuring station in the valley, rather than by personalized monitoring. This is an imperfect measurement of individual exposure due to individual variability in time spent indoors and geographical variations in ambient pollutant levels. However, the geography of the Salt Lake Valley and the pattern of weather inversions result in relatively homogenous pollution exposure for those living in this valley. We did not assess for association with specific levels of ozone, but rather extremes above the EPA National Ambient Air Quality Standards. EPA thresholds are defined based on ambient ozone pollution levels found to have health effects in epidemiologic studies, suggesting that these levels are indicative of overall exposure, despite variation in time spent in doors or out of doors. Similarly, we did not specifically address other criteria pollutants, temperature or other weather variables, which could have also had an effect on airway inflammation. However, peak levels of other criteria pollutants remained well below the EPA NAAQS during the period of our study, and were not higher on pollution testing days. With great consistency, ozone is the primary air pollutant during the summer and our indicators do reflect the real-life conditions experienced by residents in the Salt Lake Valley.

## 5. Conclusions

In this small study we found that former smokers both with and without airflow obstruction developed airway inflammation in association with short-term ozone air pollution exposure. We speculate that in susceptible individuals with COPD, airway inflammation triggered by ozone air pollution episodes may have the potential to provoke acute exacerbations of COPD or contribute to disease progression.

## References

[1] Jerrett M., Burnett R.T., Pope C.A., Ito K., Thurston G., Krewski D., Shi Y., Calle E., Thun M. (2009). Long-term ozone exposure and mortality. N. Engl. J. Med..

[2] Zanobetti A., Schwartz J. (2011). Ozone and survival in four cohorts with potentially predisposing diseases. Am. J. Respir. Crit. Care Med..

[3] Rice M.B., Ljungman P.L., Wilker E.H., Gold D.R., Schwartz J.D., Koutrakis P., Washko G.R., O’Connor G.T., Mittleman M.A. (2013). Short-term exposure to air pollution and lung function in the framingham heart study. Am. J. Respir. Crit. Care Med..

[4] Medina-Ramon M. (2006). The effect of ozone and PM_10_ on hospital admissions for pneumonia and chronic obstructive pulmonary disease: A national multicity study. Am. J. Epidemiol..

[5] Ko F.W.S., Tam W., Wong T.W., Chan D.P.S., Tung A.H., Lai C.K.W., Hui D.S.C. (2007). Temporal relationship between air pollutants and hospital admissions for chronic obstructive pulmonary disease in Hong Kong. Thorax.

[6] Halonen J.I., Lanki T., Tiittanen P., Niemi J.V., Loh M., Pekkanen J. (2010). Ozone and cause-specific cardiorespiratory morbidity and mortality. J. Epidemiol. Community Health.

[7] Arbex M.A., de Souza Conceição G.M., Cendon S.P., Arbex F.F., Lopes A.C., Moysés E.P., Santiago S.L., Saldiva P.H.N., Pereira L.A.A., Braga A.L.F. (2009). Urban air pollution and chronic obstructive pulmonary disease-related emergency department visits. J. Epidemiol. Community Health.

[8] Lee W., Thomas P.S. (2009). Oxidative stress in COPD and Its measurement through exhaled breath condensate. Clin. Transl. Sci..

[9] Yang W., Omaye S.T. (2009). Air pollutants, oxidative stress and human health. Mutat. Res./Genet. Toxicol. Environ. Mutagen..

[10] Rihák V., Zatloukal P., Chládková J., Zimulová A., Havlínová Z., Chládek J. (2010). Nitrite in exhaled breath condensate as a marker of nitrossative stress in the airways of patients with asthma, COPD, and idiopathic pulmonary fibrosis. J. Clin. Lab. Anal..

[11] Gessner C., Hammerschmidt S., Kuhn H., Hoheisel G., Gillissen A., Sack U., Wirtz H. (2007). Breath condensate nitrite correlates with hyperinflation in chronic obstructive pulmonary disease. Respir. Med..

[12] Kostikas K. (2003). Oxidative stress in expired breath condensate of patients with COPD. Chest.

[13] Biernacki W.A., Kharitonov S.A., Barnes P.J. (2003). Increased leukotriene B4 and 8-isoprostane in exhaled breath condensate of patients with exacerbations of COPD. Thorax.

[14] Huang W., Wang G., Lu S.-E., Kipen H., Wang Y., Hu M., Lin W., Rich D., Ohman-Strickland P., Diehl S.R. (2012). Inflammatory and oxidative stress responses of healthy young adults to changes in air quality during the Beijing Olympics. Am. J. Respir. Crit. Care Med..

[15] Manney S., Meddings C.M., Harrison R.M., Mansur A.H., Karakatsani A., Analitis A., Katsouyanni K., Perifanou D., Kavouras I.G., Kotronarou N. (2012). Association between exhaled breath condensate nitrate + nitrite levels with ambient coarse particle exposure in subjects with airways disease. Occup. Environ. Med..

[16] Morris A.H., Kanner R.E., Crapo R.O., Gardner R.M. (1984). Clinical Pulmonary Function Testing: A Manual of Uniform Laboratory Procedures.

[17] Hurst J.R., Vestbo J., Anzueto A., Locantore N., Müllerova H., Tal-Singer R., Miller B., Lomas D.A., Agusti A., MacNee W. (2010). Evaluation of COPD Longitudinally to Identify Predictive Surrogate Endpoints (ECLIPSE) investigators: Susceptibility to exacerbation in chronic obstructive pulmonary disease. N Engl J Med.

[18] Utah DEQ: DAQ. http://www.airquality.utah.gov.

[19] Faustini A., Stafoggia M., Cappai G., Forastiere F. (2012). Short-term effects of air pollution in a cohort of patients with chronic obstructive pulmonary disease. Epidemiology.

[20] Bell M.L., McDermott A., Zeger S.L., Samet J.M., Dominici F. (2004). Ozone and short-term mortality in 95 US urban communities, 1987–2000. JAMA.

[21] Gold D.R., Damokosh A.I., Pope C.A., Dockery D.W., McDonnell W.F., Serrano P., Retama A., Castillejos M. (1999). Particulate and ozone pollutant effects on the respiratory function of children in southwest Mexico City. Epidemiology.

[22] Higgins B.G., Francis H.C., Yates C.J., Warburton C.J., Fletcher A.M., Reid J.A., Pickering C.A., Woodcock A.A. (1995). Effects of air pollution on symptoms and peak expiratory flow measurements in subjects with obstructive airways disease. Thorax.

[23] Respiratory Research. http://www.respiratoryresearch.com.

[24] Verbeke G., Molenberghs G. (2009). Linear Mixed Models for Longitudinal Data.

[25] Alfaro M.F., Walby W.F., Adams W.C., Schelegle E.S. (2007). Breath condensate levels of 8-isoprostane and leukotriene B4 after ozone inhalation are greater in sensitive *versus* nonsensitive subjects. Exp. Lung Res..

[26] Nightingale J.A., Rogers D.F., Barnes P.J. (1999). Effect of inhaled ozone on exhaled nitric oxide, pulmonary function, and induced sputum in normal and asthmatic subjects. Thorax.

[27] Nightingale J.A., Rogers D.F., Fan Chung K., Barnes P.J. (2000). No effect of inhaled budesonide on the response to inhaled ozone in normal subjects. Am. J. Respir. Crit. Care Med..

[28] Barraza-Villarreal A., Sunyer J., Hernandez-Cadena L., Escamilla-Nuñez M.C., Sienra-Monge J.J., Ramírez-Aguilar M., Cortez-Lugo M., Holguin F., Diaz-Sánchez D., Olin A.C. (2008). Air Pollution, airway inflammation, and lung function in a cohort study of Mexico city schoolchildren. Environ. Health Perspect..

[29] Liu L., Poon R., Chen L., Frescura A.-M., Montuschi P., Ciabattoni G., Wheeler A., Dales R. (2009). Acute effects of air pollution on pulmonary function, airway inflammation, and oxidative stress in asthmatic children. Environ. Health Perspect..

[30] Ito K., de Leon S.F., Lippmann M. (2005). Associations between ozone and daily mortality: Analysis and meta-analysis. Epidemiology.

[31] Levy J.I., Chemerynski S.M., Sarnat J.A. (2005). Ozone exposure and mortality: An empiric bayes metaregression analysis. Epidemiology.

[32] Zanobetti A., Schwartz J. (2008). Mortality displacement in the association of ozone with mortality: An analysis of 48 cities in the United States. Am. J. Respir. Crit. Care Med..

[33] Peng R.D., Samoli E., Pham L., Dominici F., Touloumi G., Ramsay T., Burnett R.T., Krewski D., le Tertre A., Cohen A. (2013). Acute effects of ambient ozone on mortality in Europe and North America: Results from the APHENA study. Air Qual. Atmos. Health.

[34] Medina-Ramón M., Schwartz J. (2008). Who is more vulnerable to die from ozone air pollution?. Epidemiology.

[35] Sunyer J., Basagaña X. (2001). Particles, and not gases, are associated with the risk of death in patients with chronic obstructive pulmonary disease. Int. J. Epidemiol..

[36] Liu J., Sandrini A., Thurston M.C., Yates D.H., Thomas P.S. (2007). Nitric oxide and exhaled breath nitrite/nitrates in chronic obstructive pulmonary disease patients. Respiration.

[37] Peacock J.L., Anderson H.R., Bremner S.A., Marston L., Seemungal T.A., Strachan D.P., Wedzicha J.A. (2011). Outdoor air pollution and respiratory health in patients with COPD. Thorax.

